# Sick and Wounded in Confederate Pens and Prisons

**Published:** 1865-03

**Authors:** 


### SICK AND WOUNDED IN CONFEDERATE PENS AND PRISONS.

The inhuman treatment our soldiers have received while prisoners in the hands of the rebels, has become so outrageous and unendurable that a proposition has been brought forward in Congress, to retaliate upon the rebel prisoners in our hands, and treat them in the same inhuman manner. It will not of course be adopted, and whatever the provocation, could not be imitated by any civilized people. The evidence of the barbarism extended to our sick prisoners has accumulated until there is no longer ground for doubt that the privations and tortures which have been borne by our sick while in rebel hands, is awful beyond all conception. The rebel government in the treatment of those who were unfortunate enough to fall into their hands, has shown itself barbarian and bruitish beyond all parallel, and no deeds of valor or heroism can redeem such a government and people from damnable infamy. That the rebels have faught like heroes—have suffered, and bled, and died for their cause, impartial history will record; the same pen will also write infamy, barbarism, brutality, and all the damnable, dastardly deeds of damnation right over their crowning records of glory and valor. If they have fought like heroes, have shown devotion and self-sacrifice worthy a better cause, they have also acted like devils, and their heroic exploits are stained with the meanest and most contemptible crimes.

We have been accustomed to respect in some degree the Southern character, as presented in the more aristocratic and high-minded, but the developments of this great struggle have a thousand times made us wish that the whole Southern Confederacy—the places, names and deeds, which must forever remain one of the darkest records of American history, might be blotted from the earth and forgotten. To read the privations, sufferings, tortures and starvations inflicted upon our soldiers in Southern pens and prisons, makes us hate and despise the people as a race, and look upon them as a breed of hounds, too ugly and too mean for earth, who should have some place by themselves—some place which has no name, no way leading to it or opening from it. *Eleven thousand victims* have been buried uncoffined in the shallow trenches near Andersonville prison alone, the reports of the surgeons showing conclusively that they were unclothed, unfed, unsheltered, inhumanly and barbarously neglected, and after death almost uncovered, a record over which the very devils might have blushed and wept. To apologize for such crimes, by saying that the people were themselves unprovided with food, is only to add, if possible, to the guilt. If they could not feed, they should not confine and imprison. There can be no excuse for this unnamable inhumanity.

September 5th, Dr. J. C. Pelot reports as follows: "I would earnestly call your attention to the article of diet. The corn-bread received from the bakery being made up without sifting, *is wholly*

*unfit for the use of the sick, and often (as in the last twenty-four hours) upon examination, the inner portion is found to be perfectly raw.* The meat (beef) received by the patients, does not amount to over two ounces per day, and for the past two or three days no flour has been issued to the sick. The corn-bread cannot be eaten by many, for to do so would be to increase the disease of the bowels from which a large majority are suffering, and it is therefore thrown away. *All, then, that is received by way of sustenance is two ounces of boiled beef, and one-half pint of rice soup per day. Under these circumstances, all the skill that can be brought to bear on the case by the medical officer will avail nothing.*"

It is unnecessary to repeat or quote the evidence, and we have no heart to re-produce it; we turn away and would gladly close our eyes, and shut our ears, and if possible forget that such facts were left to shame creation. We have referred only to the care of the sick and wounded; the robust and the strong have suffered equally with the sick, and the record of mortality shows that no one could long endure such suffering and privation.

It should not be omitted, however, in connection with this painful subject, to say that the medical officers have, as a rule, been attentive and kind, doing all within their reach for the relief of these poor victims of deluded savages, who would revenge their fancied wrongs upon the sick and helpless. This makes us proud of a profession, which has done so much, and done it so impartially, for the relief of human suffering. We have nowhere seen it appear that physicians failed to do their duty, so far as circumstances would permit, and the reports from medical officers, are convincing that they did what they could, and faithfully called upon their government to provide for those under their care.

